# Deficiency in CD4 T Cells Leads to Enhanced Postpartum Internal Carotid Artery Vasoconstriction in Mice: The Role of Nitric Oxide

**DOI:** 10.3389/fphys.2021.686429

**Published:** 2021-06-16

**Authors:** Natalia I. Gokina, Rebecca I. Fairchild, Kirtika Prakash, Nicole M. DeLance, Elizabeth A. Bonney

**Affiliations:** ^1^Department of Obstetrics, Gynecology and Reproductive Sciences, Larner College of Medicine, The University of Vermont, Burlington, VT, United States; ^2^Microscopy Imaging Center, Larner College of Medicine, The University of Vermont, Burlington, VT, United States

**Keywords:** CD4 T lymphocyte, postpartum, reproductive immunology maternal physiology, eNOS, vasocontriction, vasodilation, arterial remodeling

## Abstract

The risk of postpartum (PP) stroke is increased in complicated pregnancies. Deficiency in CD4 T cell subsets is associated with preeclampsia and may contribute to PP vascular disease, including internal carotid artery (ICA) stenosis and stroke. We hypothesized that CD4 T cell deficiency in pregnancy would result in ICA dysregulation, including enhanced ICA vasoconstriction. We characterized the function, mechanical behavior, and structure of ICAs from C57BL/6 (WT) and CD4 deficient (CD4KO) mice, and assessed the role of NO in the control of ICA function at pre-conception and PP. WT and CD4KO mice were housed under pathogen-free conditions, mated to same-strain males, and allowed to litter or left virgin. At 3 days or 4 weeks PP, mice were euthanized. The responses to phenylephrine (PE), high K^+^ and acetylcholine (ACh) were assessed in pressurized ICAs before and after NOS inhibition. Passive lumen diameters were measured at 3–140 mmHg. eNOS and iNOS expression as well as the presence of T cells were evaluated by immunohistochemistry. Constriction of WT ICAs to PE was not modified PP. In contrast, responses to PE were significantly increased in ICAs from PP as compared to virgin CD4KO mice. Constriction to high K^+^ was not enhanced PP. ICAs from WT and CD4KO mice were equally sensitive to ACh with a significant rightward shift of dose-response curves after L-NNA treatment. NOS inhibition enhanced PE constriction of ICAs from WT virgin and PP mice. Although a similar effect was detected in ICAs of virgin CD4KO mice, no such changes were observed in vessels from PP CD4KO mice. Passive arterial distensibility at physiological levels of pressure was not modified at PP. ICA diameters were significantly increased in PP with no change in vascular wall thickness. Comparison of eNOS expression in virgin, 3 days and 4 weeks PP revealed a reduced expression in ICA from CD4 KO vs. WT PP vessels which reached significance at 4 weeks PP. iNos expression was similar and decreased over the PP period in vessels from WT and CD4KO mice. Dysregulation of the CD4 T cell population in pregnancy may make ICA vulnerable to vasospasm due to decreased NO-dependent control of ICA constriction. This may lead to cerebral hypoperfusion and increase the risk of maternal PP stroke.

## Introduction

Normal pregnancy manifests metabolic, immune and cardiovascular maternal adaptations that are important for successful fetal development and women’s health (Bernstein et al., 1998; Thornburg et al., 2000; Roberts and Hubel, 2010; Brien et al., 2019). The most dramatic changes occur in the maternal uterine circulation, including remarkable growth of uteroplacental vasculature and enhanced uterine vasodilation (Thornburg et al., 2000; Osol and Mandala, 2009). Systemic vascular distensibility and vasodilation show a significant increase in order to accommodate maternal blood volume expansion during normal pregnancy (Magness et al., 1996; Magness, 1999; Bernstein et al., 2001). While the majority of maternal cardiovascular changes are reversible after delivery, some continue in the postpartum period (PP) and thus may modify cardiovascular risk in future pregnancies (Clapp and Capeless, 1997; Morris et al., 2015; Staff et al., 2016; Bonney et al., 2017; Gokina et al., 2021).

Observations in humans suggest that a number of pregnancy-associated disorders precede elevated risk of cardiovascular complications later in life (Aardenburg et al., 2003; Williams, 2003; Bellamy et al., 2007; Carpenter, 2007; Roberts and Hubel, 2010; Evans et al., 2011; Leslie and Briggs, 2016; Staff et al., 2016). Recently, the American Heart Association has proposed to consider preeclampsia, gestational diabetes mellitus and delivery of a growth-restricted child as the pregnancy-related risk factors for future cardiovascular disease (Mosca et al., 2011). Although pregnancy is considered as a screening test for later life health, it remains uncertain whether abnormal pregnancy causes or reveals underlying cardiovascular disease (Roberts and Hubel, 2010; Staff et al., 2016). Pre-pregnancy hypertension, obesity, diabetes or renal disease are strongly associated with an increased risk for preeclampsia during pregnancy (Aardenburg et al., 2003; Williams, 2003; Roberts and Hubel, 2010; Morris et al., 2015; Staff et al., 2016). Moreover, in animal models, there is evidence for the link between reduced uteroplacental vascular perfusion and PP vascular dysfunction (Pruthi et al., 2015; Brennan et al., 2016).

Current epidemiologic studies demonstrate that PP is associated with a significantly increased risk of stroke in pregnancies complicated by preeclampsia, hypertension, gestational diabetes or obesity (Kittner et al., 1996; Skidmore et al., 2001; Staff et al., 2016; Cheng et al., 2017). The exact cause of PP stroke remains unknown. However, significant PP cardiovascular changes including reduced blood volume, increased blood pressure, and systemic vasoconstriction (Kittner et al., 1996; Skidmore et al., 2001) may contribute to its occurrence. Clinical studies show that chronic cerebral hypoperfusion is one of the casual factors of ischemic stroke or vascular cognitive impairment (Dong et al., 2018). There is evidence that temporal cerebrovascular ischemia and visual disturbances are linked to a transient vasoconstriction of ICAs (Arning et al., 1998). In the general population, moyamoya disease is characterized by carotid vasospasm that predisposes to ischemic stroke (Miyakoshi et al., 2009). Therefore, maternal ischemic stroke may occur as a consequence of ICA occlusion due to arterial dissection or spontaneous constriction in Moyamoya disease (Miyakoshi et al., 2009; Akamatsu et al., 2014; Simon et al., 2015). Taken together, these data suggests that some pregnancy-induced changes in the function and/or structure of internal carotid arteries may contribute to cerebral ischemia and/or stroke in very late gestation or during the PP period.

The fine balance between maternal tolerance of the fetus and immune function is protective for both fetus and mother and underlies successful pregnancy. Within the maternal immune system during pregnancy, there is both homeostatic, non-fetal antigen specific expansion and contraction of several immune cell types including T cells (Norton et al., 2009, 2010). In the setting of pregnancy, global deficiency of CD4 T cells may open niches for CD8 T cell homeostatic proliferation, which has been associated with increased expression of cytotoxic molecules, such as granzyme A (Bhat et al., 2017; Fortner et al., 2017). CD4 T cells are the master regulators of adaptive immunity through productive interaction with other immune cell subsets, e.g., CD8 T cells or B cells, and lack of CD4 T cell help or collaboration may alter the function of these cells (Guerder and Matzinger, 1992). Deficiency in subsets of regulatory CD4 T cells has been associated with vascular disease (Ait-Oufella et al., 2006; Kvakan et al., 2009; Kassan et al., 2013) but may play a complex role in modulation of cerebrovascular beds (Kleinschnitz et al., 2013). It is presumed that this deficiency leads to the over activation of cytotoxic effector CD4 (Hermansson et al., 2010) and CD8 (Walch et al., 2013) T cells which may then enter the vascular wall (Gewaltig et al., 2008). Moreover, cross sectional studies in populations of pregnant women have suggested an association between deficiency in regulatory T cell subsets and preeclampsia (Cerdeira et al., 2012; Przybyl et al., 2015) or preterm birth (Kouckı et al., 2014; Gomez-Lopez et al., 2016).

Multiple molecules govern tissue-specific T cell-endothelial interaction, including trafficking molecules such as inter-cellular adhesion molecule 1 (ICAM-1) (Bharadwaj et al., 2013) CXCL1 (Sacre et al., 2012), regulated on activation normal T cell expressed and secreted (RANTES) (Guzik et al., 2007), L-selectin (Galkina et al., 2006) and others, while some endothelial molecules regulate T cell activation, e.g., MHC, (Pober et al., 2017) CD80/CD28 (Zhang et al., 2019), CD86 (Zhao et al., 2020) or effector function, e.g., Transforming growth factor beta (Lebastchi et al., 2011). Other molecular interactions, e.g., CD40/CD40L (Hausding et al., 2013), NOTCH receptor and its ligands, Jagged1 and Delta1 (Piggott et al., 2011), and Programmed Death-1(PD-1)/PD-L1 (Zhang et al., 2017) may regulate reciprocal activation. Products of activated T cells, for example IL-17, can effect change in endothelial cell function through regulation of NO (Caillon et al., 2016). Activated T cells in the vessel wall may generate reactive oxygen species [ROS, (Peng et al., 2021)], which can cause endothelial cell dysfunction or death (Park, 2013). Modulatory interactions between T cells and vascular smooth muscle cells, e.g., through FAS and FAS ligand (Jiang et al., 2004) and other molecules may also occur. Such interactions may also lead to altered vascular function either directly, or via myoendothelial feedback-induced alteration in endothelial NO (Kerr et al., 2012). CD4 T cells may regulate other immune cells, such as macrophages that can modify vascular extracellular matrix which, in addition to affecting vascular structure, may also enhance T cell infiltration (Watanabe et al., 2018). Within vascular adventitia, expression of molecules such as IL-33 that can modify T cell effector function (Li et al., 2019). We have observed in a mouse model that the absence of CD4 T cells results in an altered postpartum phenotype, particularly with regard to the response to a vasodilatory molecule, in systemic resistance vessels (Bonney et al., 2017). These data suggests a link between pregnancy-associated vascular changes and maternal T cell function.

Despite clinical and experimental evidence for the role of ICA stenosis in initiating cerebrovascular disorders, the effect of pregnancy on their function and structure remains unexplored. Moreover, it is unknown whether CD4 T cell immune deficiency can modify vascular reactivity of ICA in the PP period. We chose to test the hypothesis that *compared to the virgin/pre-pregnancy state, vessels from WT mice behave qualitatively differently than vessels from CD4 deficient mice.* Therefore, the objectives of the current study were: (1) to characterize the effect of normal pregnancy on the vasodilator and vasoconstrictor reactivity of mouse ICAs at 3 days (early PP) and 4 weeks PP (late PP); (2) to study the effect of CD4 T cell deficiency on PP ICA function and structure; (3) to delineate the role of vascular NO system in the control of ICA function in WT and CD4KO mice; and (4) to define whether these two PP periods are associated with changes in mechanical behavior and/or structure of ICAs.

## Materials and Methods

### Animals

All experimental protocols were approved by the Institutional Animal Care and Use Committee of The University of Vermont (protocol X0-140). These studies used C57BL/6J wild-type (WT, *n* = 40) from Jackson Laboratory, United States (Cat # 000664) and CD4 T cell deficient mice (CD4KO, *n* = 31) (Rahemtulla et al., 1991) also from Jackson Laboratory (Cat #002663). The CD4 KO mice used in these studies have no CD4 T cells and thus have significantly reduced helper T cell function, yet have normal myeloid cell and CD8 T cell numbers and function. Male and female mice were housed at The University of Vermont animal care facility. Females 4–6 months of age were either left virginal or mated to same-strain males. Maternal stroke is the most frequent during first 2 weeks after delivery and the risk of stroke remains elevated for 1–2 years PP period (PP) (Cheng et al., 2017; Wu P. et al., 2020). Therefore, we chose early, e.g., 3 days and late, e.g., 4 weeks after delivery of a litter as time points. Moreover, we have observed these time points as being relevant for PP mesenteric remodeling in this mouse model. Mice age, weights, and number of born pups for all studied groups are shown in Supplementary Figure 1.

### Preparation of Internal Carotid Arteries for Experimentation

On the experimental day, mice were euthanized using CO_2_ and thoracotomy. Left and right ICAs were identified, dissected out, and carefully cleaned free of perivascular connective and adipose tissue under a dissecting microscope in pre-aerated (5% O_2_, 10% CO_2_, and 85% N_2_) physiologic salt solution (PSS). Arterial segments were cannulated from both ends in a pressure arteriograph and placed on the stage of an inverted microscope with an attached video camera. Arteries were initially pressurized at 10 mmHg using a pressure servo controller system (Living Systems Instrumentation, Burlington, VT, United States) and continuously super-fused at 3 mL/min with aerated PSS at 37°C and pH = 7.4 for a 1 h equilibration period before starting experimental protocols.

### Experimental Protocols

After the equilibration period, intraluminal pressure was elevated from 10 to 80 mmHg. Following stabilization of the lumen diameter, PE was tested in increasing concentrations (0.01–30 μM). Each concentration was added for 5–7 min until stabilization of the constriction. To assess ICA endothelial function, ACh (0.01–10 μM) was applied in a cumulative fashion to arteries pre-constricted with PE to 50–60% of the initial diameter. A combination of papaverine (100 μM) and diltiazem (10 μM) was applied at the end of each experiment to fully dilate the artery.

In an additional set of experiments, we explored the effects of pregnancy and CD4 T cell immune deficiency on receptor-independent vascular smooth muscle (VSM) contraction. Vasoconstriction in response to gradual depolarization with high potassium (high K^+^) solutions (20–100 mM, 10 min for each concentration) was tested in pressurized ICAs from all studied groups of mice. To assess the role of nitric oxide (NO) in the modulation of ICA vascular reactivity, the responses of arteries from virgin, 3 days, and 4 weeks PP mice to PE, high K^+^ and ACh were evaluated after 20 min pretreatment with 200 μM of N^G^-nitro-L-arginine, L-NNA, a nitric oxide synthase (NOS) inhibitor.

Changes in the arterial diameter were continuously monitored and recorded using the IonOptix program (IonOptix LLC, Westwood, MA, United States). Lumen diameters were measured during the last 15–20 s of each tested concentration of drugs using IonOptix software. Responses to PE were expressed as the percentage of the initial arterial diameter. ACh-induced dilatation was expressed as the percentage of maximal dilator response to the application of diltiazem and papaverine. Concentration-dependent vasoconstriction or vasodilation was also expressed as the percentage of maximal response to PE, high K^+^ and ACh to define EC_50_ values. Data were imported into SigmaPlot program to construct the concentration-response curves for high K^+^-, PE-induced vasoconstriction, and ACh-induced vasodilatation.

### Vascular Distensibility and Remodeling

To characterize the effects of pregnancy and CD4 T cell deficiency on the structure and mechanical behavior of ICAs, we compared passive arterial diameters, wall thickness and distensibility of the vessels from WT and CD4KO virgin and PP mice. The arteries were pressurized at 3 mmHg and superfused for 10 min with PSS containing 20 μM diltiazem and 50 μM papaverine to inhibit vascular contractility and to allow for maximal arterial dilation at each level of intraluminal pressure. Inner (lumen, D_*in*_) and outer (D_*out*_) arterial diameters were defined after stepwise elevation in intraluminal pressure from 3 to 120 mm Hg. Three mmHg is the minimal pressure for an un-stretched vessel that prevented the vessel from collapsing. D_*out*_ and D_*in*_ were both measured from saved images of pressurized arteries on the monitor screen after stabilization of the diameters at each specific level of pressure (typically, 2–3 min for each pressure step). Arterial wall thickness (t) was calculated as follows: *t* = (D_*out*_ – D_*in*_)/2. Arterial distensibility was defined as an increment in the lumen diameter in response to pressure elevation from 3 to 120 mmHg. The increments in the lumen diameter were expressed as the percentage of an un-stretched vessel diameter at 3 mmHg.

### Immuno-Histological Examination of eNOS and iNOS Expression

Mouse ICAs pressurized at 80 mmHg were fixed in 4% paraformaldehyde for 1 h and stored at 4°C prior to paraffin embedding. 3 mm thick sections were cut on a Leica RM2145 paraffin microtome (Leica Microsystems, Buffalo Grove, IL, United States) and retrieved onto slides. Slides were air dried overnight and baked at 60°C for 1 h prior to staining. Sections were deparaffinized in three changes of xylene and rehydrated through graded ethanol. For immunofluorescence, antigen retrieval was performed using DAKO Target Retrieval Solution (pH 6.0) at 96°C, where after slides were blocked in 5% BSA/10% normal goat serum. Rabbit polyclonal anti-iNOS (Invitrogen #PA3-030A) and mouse monoclonal anti-eNOS (Abcam #ab76198) were applied to vessels at 1:400 and 1:500, respectively, overnight at 4°C, followed by rinses with buffer. The presence of bound primary antibody was detected using Invitrogen goat anti-rabbit IgG Alexa Fluor 647 secondary antibody, and goat anti-mouse IgG Alexa Fluor 555 secondary antibody. Finally, the nuclei were counterstained with DAPI and cover-slipped in aqueous mounting media. Sections were imaged on a Nikon A1R-ER Confocal Microscope using a Plan Fluor 40x Oil DIC H N2, NA 1.3, WD = 240 μm objective lens.

Image Analysis was performed using NIS-Elements Ar (version 4.30.02; Nikon, United States, Tokyo, Japan). Semi-quantitative pixel values were obtained by drawing regions of interest around the endothelial and smooth muscle layers of each vessel. Intensity thresholds for immunoreactivity were determined through evaluation of secondary antibody control images. For consistency, the thresholding value was maintained for each antibody, separately, across all mouse ICAs.

### Immune Cell Detection in the Wall of Internal Carotid Arteries

In an additional set of experiments, mouse ICAs were cleaned from connective tissue, cut-opened and pinned in a small sylgard-filled dissecting dish. Arteries were fixed in 4% paraformaldehyde for 2 h at 4°C and stored in 0.1M Phosphate Buffered Saline, pH 7.2, until use. For immunofluorescence, vessels were blocked in 5% BSA/10% normal goat serum/0.1% Triton X-100. Ready to use rabbit polyclonal CD3 (Thermo Fisher Scientific #RM-9107-R7), mouse monoclonal CD8 pre-conjugated to Alexa Fluor 488 (Invitrogen #53-0081-82) and rat monoclonal CD4 (Thermo Fisher Scientific #14-9766-82) were applied to vessels at 10 μg/ml and 5 μg/ml, respectively, overnight at 4°C, followed by rinses with buffer. Presence of bound primary antibody was detected using Invitrogen goat anti-rabbit IgG Alexa Fluor 555 secondary antibody, and goat anti-rat IgG Alexa Fluor 647 secondary antibody. Finally, the nuclei were counterstained with DAPI and cover-slipped in aqueous mounting media. Sections were imaged on a Nikon A1R-ER Confocal Microscope using a Plan Apo λ20x, NA 0.8, WD = 1000 μm objective lens. Image Analysis was performed using NIS-Elements Ar (version 4.30.02; Nikon, United States, Tokyo, Japan). Numerical counts were obtained manually for positive CD3, CD4, and CD8 cells. Total nuclei were determined using intensity thresholds for DAPI in conjunction with manual counts.

### Western Blot Analysis

Protein expression of Anti-NFκB p52 Antibody was detected by Western blot analysis. Internal carotid arteries were collected from eight to ten mice, immediately frozen in liquid nitrogen and then kept at −80°C until the day of analysis. For protein lysate preparation, samples were placed in Pierce RIPA buffer (Thermo Fisher Scientific, Waltham, MA, United States) supplemented with Halt protease and phosphatase inhibitor cocktail (Thermo Fisher Scientific) in Lysis Matrix D tubes (MP Biomedicals, Solon, OH, United States) and homogenized using two 30-s pulses on a FastPrep-24 instrument (MP Biomedicals). Total extracted protein was determined by a BCA protein assay kit (Thermo Fisher Scientific). Protein samples (20 μg of soluble protein each) were separated using 12% SDS-PAGE and transferred to a PVDF membrane. After blocking with 5% BSA, mouse monoclonal NFκB p52 (1:1,000, Sigma-Aldrich; 05-361), mouse monoclonal β-actin (1:5,000, Sigma-Aldrich; A5441) primary antibodies and horseradish peroxidase-conjugated secondary antibody (GE Healthcare; NA934) were used to reveal specific proteins on the blot. Protein bands were detected by Super Signal West Pico chemiluminescent substrate (Thermo Fisher Scientific).

### Solutions and Drugs

The physiological salt solution (PSS) contained: 119 mM NaCl, 4.7 mM KCl, 24.0 mM NaHCO_3_, 1.2 mM KH_2_PO_4_, 1.6 mM CaCl_2_, 1.2 mM MgSO_4_, 0.023 mM EDTA, and 11.0 mM glucose, pH = 7.4. High K^+^ solutions were prepared by equimolar substitution of NaCl with KCl. All chemicals were purchased from Thermo Fisher Scientific (Agawam, MA, United States). ACh, PE, diltiazem and papaverine were obtained from Sigma Chemical Co. (St. Louis, MO, United States). Diltiazem was prepared as a 10 mM stock solution in deionized water and kept refrigerated until use (1–2 weeks). Papaverine was dissolved in deionized water on the day of the experiment. Stock solution of N^*G*^-nitro-L-arginine, L-NNA in PSS, and ACh and PE in deionized water were prepared on each experimental day.

### Statistical Analyses

Statistical analysis of functional and structural data was performed using SigmaPlot version 14 (Systat Software Inc., San Jose, CA, United States). Vasoconstrictor (PE and high K^+^) and vasodilator (ACh) responses were expressed as a percentage of initial or passive lumen diameters at 80 mmHg, respectively. Distensibility was expressed as noted above. We compared vessel response data from different groups of mice by two-way ANOVA and pairwise comparison. Data are presented as mean vascular response ± standard error of the mean at each element of exposure (e.g., concentration, pressure). Arterial sensitivity to ACh, PE and High K^+^ was determined based on EC_50_ values (the concentration that induced 50% of the effect) calculated for each tested artery using Standard Curves Analysis function of SigmaPlot program. The one way ANOVA was used to compare structural parameters (e.g., lumen diameters and wall thickness) from groups of vessels. Pixel intensities of eNOS or iNOS staining were compared by the unpaired *t* test. Vessel immune cell frequencies expression data and were compared by the Mann Whitney Test. Data were considered significantly different at *P* < 0.05.

## Results

### CD4 T Cell Deficiency Enhances Constrictor Reactivity of Postpartum Internal Carotid Arteries to Phenylephrine

**FIGURE 1:**
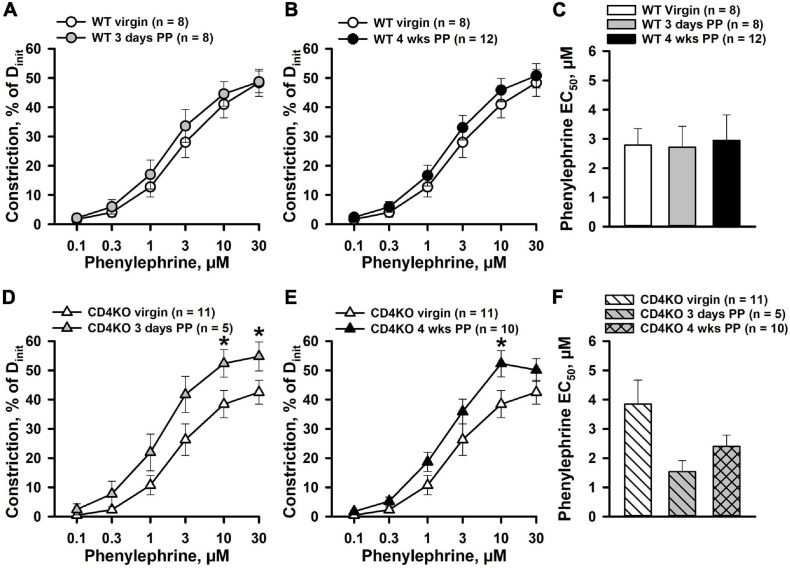
Differential effects of pregnancy on phenylephrine (PE)-induced constriction of internal carotid arteries (ICAs) from C57BL/6 wild-type (WT) and CD4 T cell deficient (CD4KO) mice. **(A,B)** PE induced a comparable concentration-dependent constriction of arteries from WT virgin, 3 days and 4 weeks postpartum (PP) WT mice (*P* > 0.05, two-way ANOVA). **(C)** A bar graph showing no difference in concentration of PE producing 50% constriction (EC_50_ values) of ICAs from virgin vs. PP mice (*P* > 0.05, one way ANOVA). **(D,E)** Enhancement of PE-induced constriction of ICAs from CD4KO 3 days and 4 weeks PP mice (*P < 0.05, two way ANOVA). **(F)** A bar graph demonstrating a trend in reduction of EC_50_ values for PE-induced constriction of ICAs from CD4KO^–^ PP mice compared to virgins (*P* > 0.05, one way ANOVA).

We tested and compared constrictor responses to PE in ICAs from virgin, 3-days and 4 weeks PP WT and CD4KO mice. No significant differences in baseline lumen diameters at 80 mmHg in PSS were observed between virgin, 3 days PP and 4 weeks PP WT and CD4KO mice (Supplementary Table 1). The application of PE resulted in concentration-dependent constriction of ICAs from 3 days and 4 weeks PP WT mice that was not different from the responses of ICAs from WT virgin mice (Figures 1A,B). No significant changes were found in EC_50_ values for PE-induced constriction in vessels from the mice studied (Figure 1C). In contrast, PE-induced constrictor responses of ICAs from 3 days and 4 weeks PP CD4KO mice were significantly increased compared to PE-induced constrictions of vessels from virgin CD4KO mice (Figures 1D,E). EC_50_ values for PE-induced constriction tended to decrease in the PP period (*P* = 0.120, Figure 1F). These data indicate that CD4 T cell deficiency in mice results in enhanced contractility of ICAs during the PP period.

### CD4 T Cell Deficiency Does Not Enhance the Constrictor Reactivity of Internal Carotid Arteries to High K^+^

**FIGURE 2:**
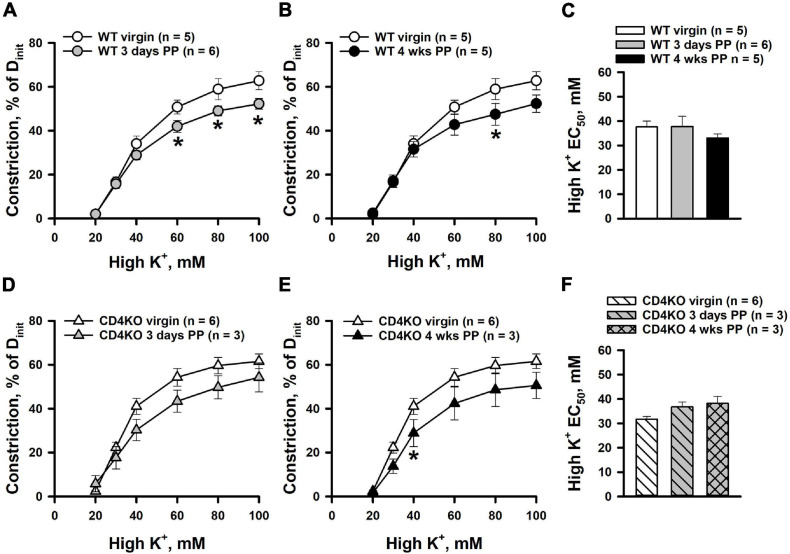
High potassium (high K^+^)-induced constrictor responses of internal carotid arteries (ICAs) from C57BL/6 wild-type (WT) and CD4 T cell deficient (CD4KO) virgin and postpartum (PP) mice **(A,B)** Summary graphs showing a reduction in high K^+^-induced constriction of arteries from 3 days and 4 weeks PP WT mice compared to virgin controls (*P < 0.05, two way ANOVA). **(C)** Unaltered EC_50_ values for high K^+^-induced constriction in arteries from WT PP and virgin mice (*P* > 0.05, one way ANOVA). **(D,E**) Constrictor responses to high K^+^ solutions were unchanged in ICAs from 3 days and 4 weeks PP CD4KO mice compared to virgin controls (*P* > 0.05, two-way RM ANOVA). **(F)** EC_50_ values for high K^+^ evoked constriction were not different in arteries from virgin vs. PP CD4KO mice (*P* > 0.05, one way ANOVA).

To determine whether CD4 T cell deficiency modifies the intrinsic vascular smooth muscle (VSM) contractility of ICAs PP, constrictor responses to non-receptor activation of VSM with graded K^+^ depolarization were studied next. Figure 2 demonstrates constrictor responses of ICAs plotted as a function of K^+^ concentration. High K^+^-induced constriction was significantly decreased in vessels from 3 days (Figure 2A) and 4 weeks WT PP (Figure 2B) compared to virgin in WT mice. However, based on EC_50_ values, sensitivity of arteries to K^+^-induced depolarization was not significantly different between WT virgin and PP groups (Figure 2C). No significant differences were found in K^+^-induced constrictor responses of ICAs from virgin vs. 3 days and 4 weeks PP CD4KO mice (Figures 2D,E). Consistent with this, EC_50_ values for high K^+^ constriction were similar in vessels from all three CD4KO groups (Figure 2F).

### CD4 T Cell Immune Deficiency Does Not Affect ACh-Induced Dilation of Internal Carotid Arteries From Virgin and Postpartum Mice

**FIGURE 3:**
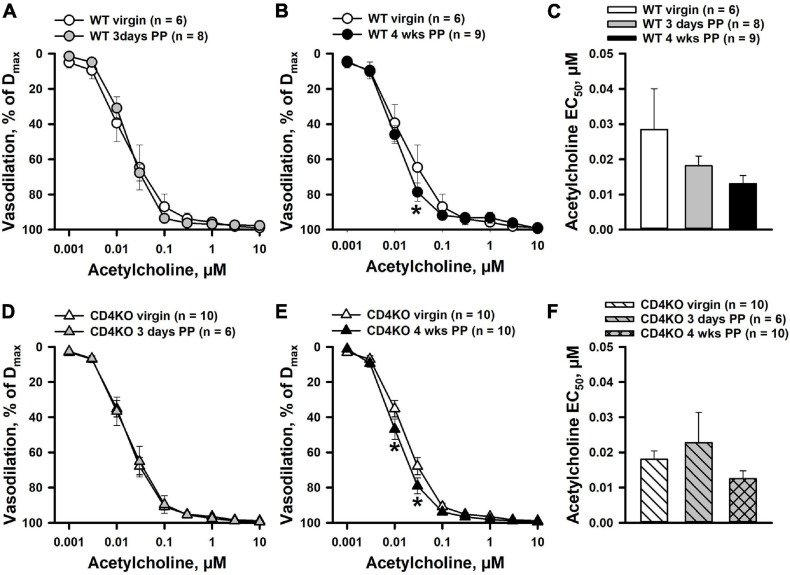
The effects of pregnancy on acetylcholine (ACh)-induced dilation of internal carotid arteries (ICAs) from C57BL/6 wild-type (WT) and CD4 T cell deficient (CD4KO) virgin and postpartum (PP) mice. **(A)** A summary graph demonstrating no difference in ACh-induced dilator responses of arteries from 3 days PP vs. virgin WT mice (*P* > 0.05, two way RM ANOVA). **(B)** Dilator responses to ACh were increased in 4 weeks PP vs. virgin WT mice (*P < 0.05, two way ANOVA). **(C)** EC_50_ values for ACh-induced vasodilation of arteries from WT PP mice were not significantly different from that of vessels from virgin controls (*P* > 0.05, one way ANOVA). **(D)** Unaltered dilator responses of ICAs to ACh in 3 days PP vs. virgin CD4KO mice (*P* > 0.05, two way ANOVA). **(E)** A summary graph showing an increased reactivity to ACh in ICAs from 4 weeks PP vs. virgin CD4KO mice (*P < 0.05, two way RM ANOVA). **(F)** A bar graph summarizing EC_50_ values for ACh-induced dilation of ICAs from virgin, 3 days and 4 weeks PP CD4KO mice. (*P* > 0.05, one way ANOVA).

The effects of pregnancy and CD4 T cell deficiency on endothelial function of ICAs were assessed by testing vascular responses to endothelium-dependent vasodilator ACh. Application of ACh to ICAs pre-constricted with PE resulted in concentration-dependent vasodilation in all vascular groups. ICAs were highly sensitive to ACh with near maximal dilatation observed at 0.1 μM. No significant differences were found in dilator responses of ICAs from 3 days PP WT (Figure 3A) or CD4KO (Figure 3D) as compared to vessels from same strain virgin mice. A modest increase in ACh-induced vasodilation was detected in arteries from both WT (Figure 3B) and CD4KO (Figure 3E) 4 weeks PP mice. EC_50_ values for ACh-induced dilation were not significantly modified PP in ICAs from either WT (Figure 3C) or CD4KO (Figure 3F) mice.

### The Role of Nitric Oxide in the Control of Internal Carotid Artery Function

**FIGURE 4:**
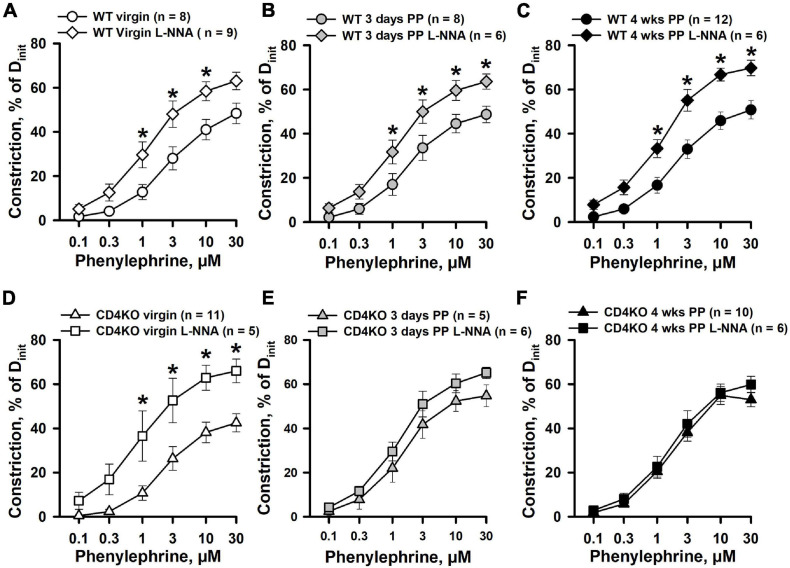
The effects of nitric oxide synthase (NOS) inhibition with L-NNA on phenylephrine (PE)-induced constrictor responses of internal carotid arteries from C57BL/6 wild-type (WT) and CD4 T cell deficient (CD4KO) virgin and postpartum (PP) mice. **(A–C)** Summary graphs showing that blockade of NO production with L-NNA resulted in a significant enhancement of PE-induced constriction of arteries from virgin **(A)**, 3 days **(B)**, and 4 weeks **(C)** PP WT mice (*P < 0.05, two way RM ANOVA). **(D)** PE-induced responses of ICAs from CD4KO virgin mice were significantly increased by L-NNA treatment (*P < 0.05, two way RM ANOVA). Inhibition of NO production with L-NNA did not alter PE-induced responses of arteries from 3 days **(E)** and 4 weeks **(F)** PP CD4KO mice (*P* > 0.05, two way RM ANOVA).

In our previous studies, mesenteric artery PE-induced constriction was significantly reduced in early PP but was restored to pre-pregnancy levels after inhibition of NO production with L-NNA (Gokina et al., 2021). These data implicate an important modulatory role of NO in regulation of PE-induced mesenteric vasoconstriction in the PP period. To clarify the role of NO in regulation of vascular contractility of ICAs during the PP period, we studied PE- and high K^+^-induced constrictor responses of arteries pre-treated with the NOS inhibitor L-NNA (200 μM). In 20 min of L-NNA application, no significant differences in baseline lumen diameters of ICAs were observed between virgin, 3 days PP and 4 weeks PP WT and CD4KO mice (Supplementary Table 2). Blockade of NO production resulted in a significant increase in PE-induced constriction of ICAs from virgin (Figure 4A) as well as 3 days (Figure 4B), and 4 weeks PP WT mice (Figure 4C). EC_50_ values for PE-induced constriction in the context of L-NNA tend to be reduced but were not significantly modified in virgin and PP mice, demonstrating no change in the sensitivity of ICAs to PE after blockade of NO production (Supplementary Figures 2A–C). As in WT vessels, L-NNA-induced potentiation of PE-driven constriction was found in ICAs from virgin CD4KO mice (Figure 4D). However, blockade of NO production resulted in no changes in PE-induced constriction of ICAs from 3 days and 4 weeks PP CD4KO mice (Figures 4E,F). EC_50_ values were reduced only in L-NNA-treated ICAs from CD4KO virgin mice (Supplementary Figure 2D). In contrast, L-NNA did not change the sensitivity to PE observed in CD4KO vessels from 3 days PP or from 4 weeks PP mice (Supplementary Figures 2E,F). This suggests that in ICAs from CD4 KO mice, basal production of NO may play a significant (e.g., in virgin vessels) role in modifying the constrictor response to PE that is diminished PP.

**FIGURE 5:**
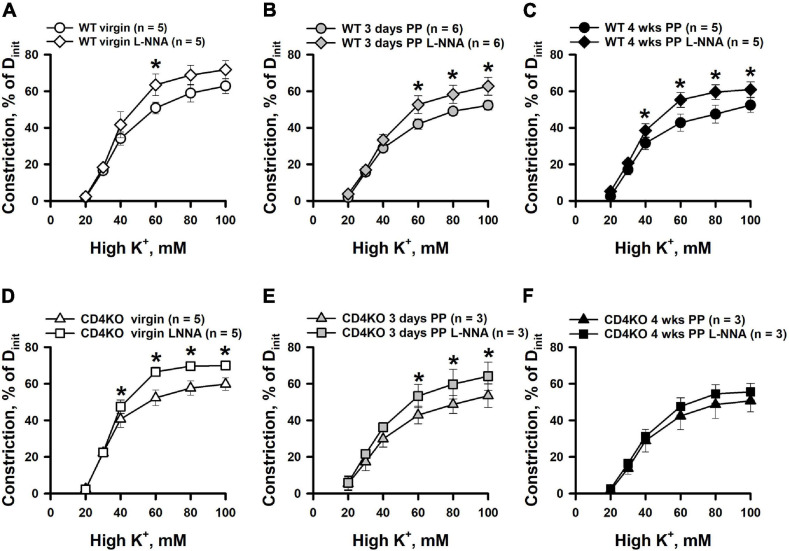
An enhancement of high K^+^ induced constriction of internal carotid arteries (ICAs) from C57BL/6 wild-type (WT) and CD4 T cell deficient (CD4KO) virgin and postpartum (PP) mice after blockade of NO production with L-NNA. **(A–C)** Summary graphs showing a significant potentiation of high K^+^ induced responses of ICAs from WT mice after inhibition of NOS activity with L-NNA (*P < 0.05, two way RM ANOVA). L-NNA treatment resulted in an increase in high K^+^-induced constriction in arteries from CD4KO virgin **(D)** and 3 days **(E)** PP mice (*P < 0.05, two way RM ANOVA). **(F)** A summary graph showing unaltered high K^+^-evoked constriction of arteries from 4 weeks PP vs. virgin CD4KO mice (*P* > 0.05, two way ANOVA).

Inhibition of NO production resulted in enhancement of high K^+^-induced constriction of ICAs from WT virgin and PP mice (Figures 5A–C). A similar enhancement of K^+^-induced constriction by L-NNA treatment was observed in ICA from virgin CD4KO mice (Figure 5D). Modest or no significant changes were detected in responses to high K^+^ solutions after NOS inhibition in ICAs from 3 days and 4 weeks CD4KO PP mice, respectively (Figures 5E,F). EC_50_ values for high K^+^-induced constriction were not significantly modified by NOS inhibition in vessels from either virgin or PP WT and CD4KO mice (Supplementary Figure 3).

**FIGURE 6:**
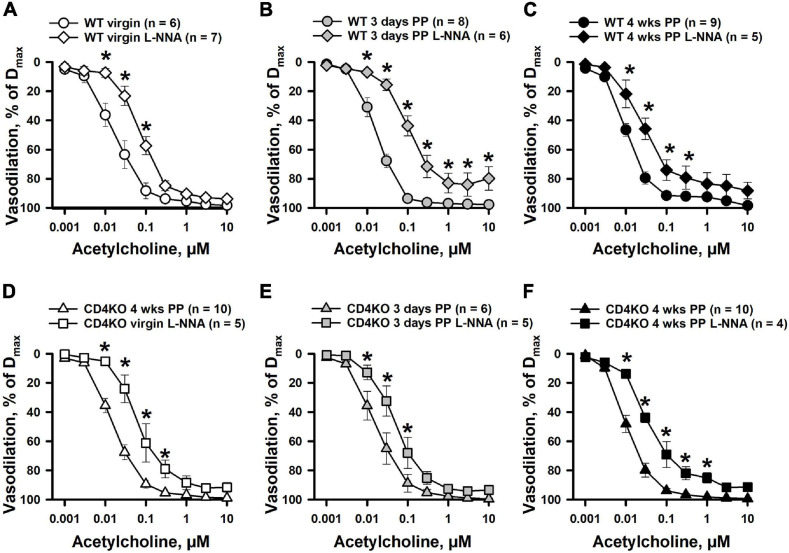
The effects of NOS inhibition with L-NNA on acetylcholine (ACh)-induced vasodilation of internal carotid arteries (ICAs) from virgin and postpartum (PP) C57BL/6 wild-type **(A–C)** and CD4KO **(D–F)** mice. In all tested groups of mice, a blockade of NO production resulted in a significant shift in ACh concentration-response curves. In the presence of L-NNA, ACh still induced near maximal dilatation of ICAs (*P < 0.05, two way ANOVA).

Next, the contribution of NO in ACh-induced vasodilation of ICAs was studied in arteries pre-treated with L-NNA. Although inhibition of NO production resulted in reduced reactivity to ACh and a marked rightward shift in concentration-response curves in all studied groups of ICAs, no changes were observed in the maximal vasodilator responses to ACh (Figure 6). Further, there was a significant increase in EC_50_ values for ACh-induced dilation of ICAs indicating reduced sensitivity of arteries to ACh after blockade of NOS (Supplementary Figure 4) that was significant in 3 days PP WT but not so in 3 days PP CD4KO vessels. In ICAs from 4 weeks PP WT mice treated with a combination of L-NNA (200 μM) and indomethacin (10 μM) to inhibit production of NO and prostacyclin, respectively, ACh can induce near maximal vasodilation (Supplementary Figure 8).

### Effects of Pregnancy and CD4 T Cell Deficiency on eNOS and iNOS Expression

It is acknowledged that NO controls VSM contractility via multiple mechanisms. Endothelial (eNOS) and inducible (iNOS) NOS are two major isoforms that are present in the blood vessel wall which modify vascular function under physiological or disease conditions (Sladek et al., 1997; Feletou and Vanhoutte, 2006; Sutton et al., 2020).

**FIGURE 7:**
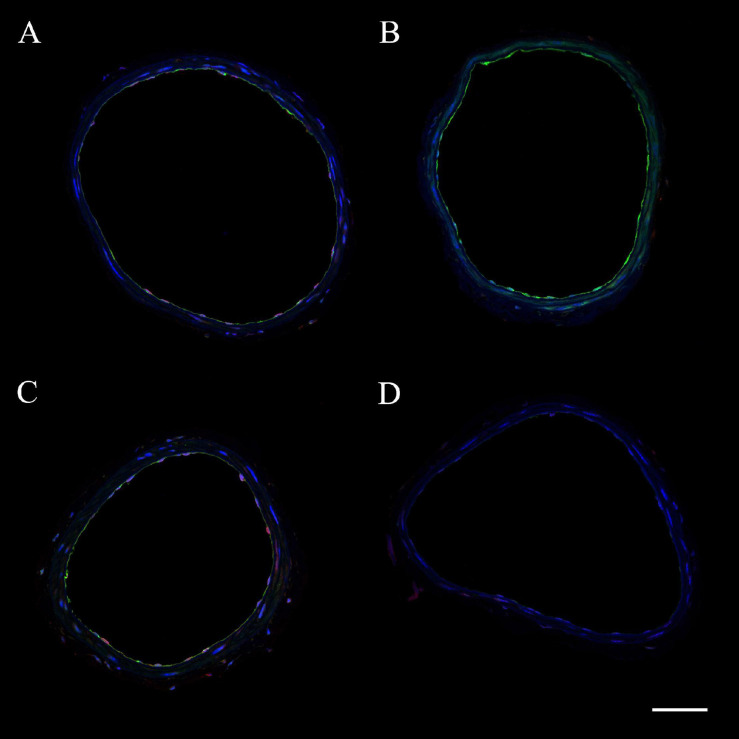
Representative images of internal carotid artery cross sections from C57BL/6 wild-type (WT) virgin **(A)**, WT 4 weeks PP **(B)**, CD4 T cell deficient (CD4KO) virgin **(C)**, and CD4KO 4 weeks PP **(D)** mice. eNOS is shown in green and iNOS – in red. Endothelial and smooth muscle cell nuclei are stained in blue (DAPI). The scale bar is 50 μm.

**FIGURE 8:**
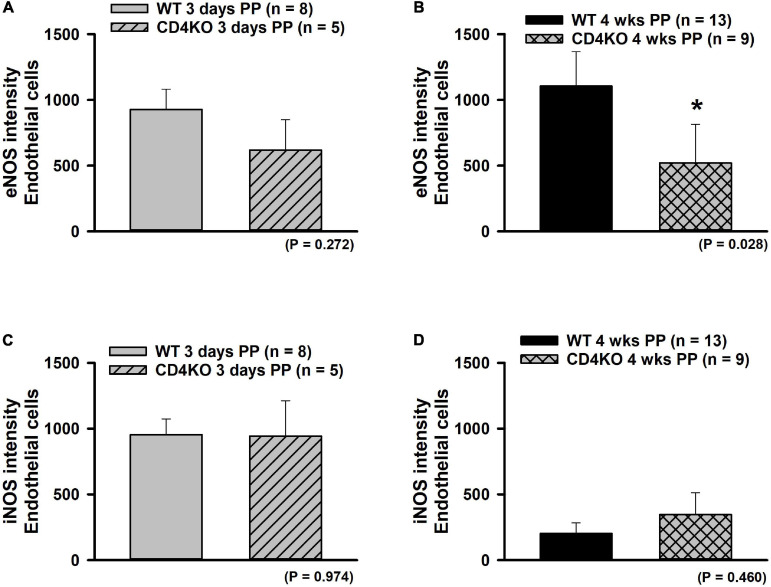
The effect of pregnancy on eNOS and iNOS expression in endothelium of internal carotid arteries (ICAs) from C57BL/6 wild-type (WT) and CD4 T cell deficient (CD4KO) mice. **(A,B)** Bar graphs summarizing the expression of eNOS in endothelial cells of arteries from WT and CD4KO PP mice. eNOS expression tends to be lower in arteries from 3 days CD4KO PP vs. 3 days PP WT mice **(A)**. This trend reached a significance at 4 weeks PP **(B)**. **(C,D)** iNOS expression was not different in arteries from WT vs. CD4KO mice at any PP period (*significantly different at *P* < 0.05, unpaired *t*-test).

The expression of eNOS or iNOS in endothelial and vascular smooth muscle wall layers was assessed in ICAs from WT and CD4KO mice based on intensities of fluorescence from ICA cross sections (Figure 7, enhanced view, Supplementary Figure 5). Endothelial eNOS expression in WT vs. CD4KO ICAs was similar at 3 days PP (Figure 8A). However, at 4 weeks PP, endothelial expression of eNOS in WT ICAs was significantly higher than in CD4KO (Figure 8B). Expression of eNOS in virgin endothelial cells of WT and CD4KO ICAs was similar (Supplementary Figure 5A).

Expression of iNOS was similar in WT compared to CD4KO endothelial cells at 3 days PP (Figure 8C) and at 4 weeks PP (Figure 8D). While iNOS endothelial cell expression decreased from 3 days to 4 weeks PP in vessels of both WT and CD4KO mice, this decrease reached significance only in vessels from WT mice (Figures 8C,D). Of note, expression of iNOS in virgin endothelial cells of WT was lower than that found in virgin CD4KO ICAs (Supplementary Figure 5B).

In vascular smooth muscle cells of WT vs. CD4KO ICAs, expression of eNOS was not different at any time point tested (Supplementary Figures 6A,B). While overall iNOS expression was similar in vascular smooth muscle cells of WT and CD4KO ICAs (Supplementary Figures 6C,D), expression of iNOS in WT, but not CD4KO smooth muscle cells significantly decreased from 3 days to 4 weeks PP. Thus, eNOS and iNOS were detectable in endothelial cells and smooth muscle cells of both WT and CD4KO mice with a trend toward differential regulation PP.

### The Effects of Pregnancy and CD4 Immune Deficiency on the Mechanical Behavior and Remodeling of Internal Carotid Arteries

**FIGURE 9:**
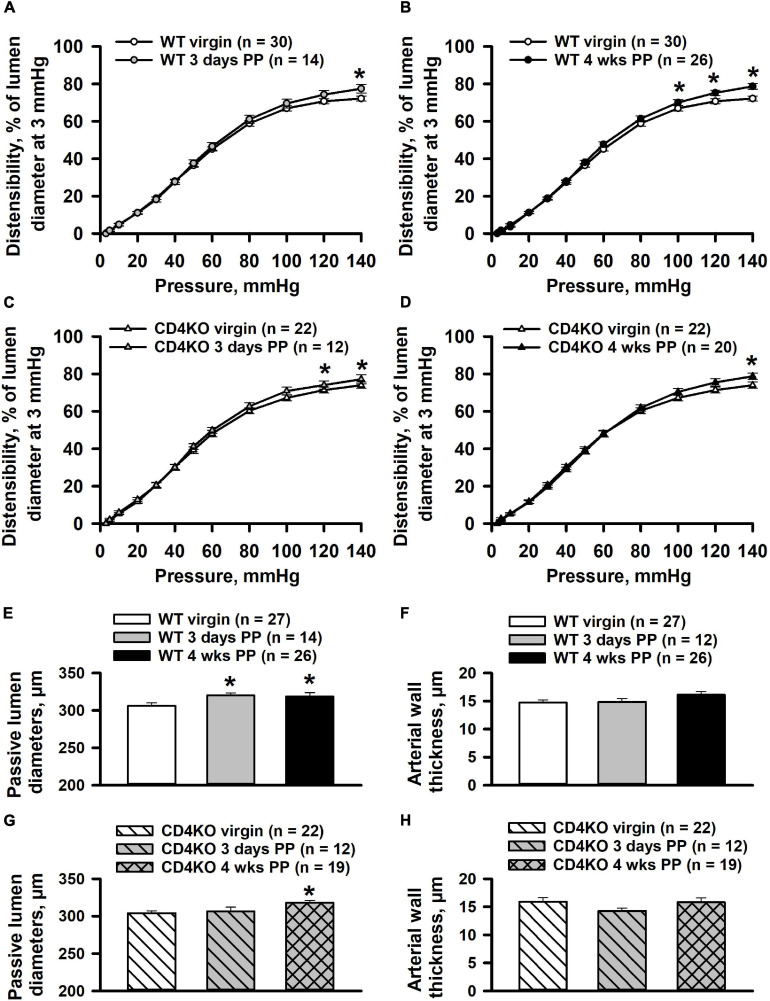
Effects of pregnancy on distensibility and structural parameters of internal carotid arteries (ICAs) from wild-type (WT) and CD4 T cell deficient (CD4KO) mice. Summary graphs showing changes in distensibility of arteries from WT **(A,B)** and CD4KO **(C,D)** mice before pregnancy (virgin), 3 days and 4 weeks postpartum (PP). Distensibility is expressed as the percentage of pressure-induced changes in passive lumen diameters relative to that of an un-stretched arteries at 3 mmHg. Significant changes in distensibility of PP arteries were observed only at high levels (100–140 mmHg) of intraluminal pressure (*significantly different at *P* < 0.05 two way ANOVA). Summary graphs showing passive lumen diameters **(E)** and wall thicknesses **(F)** of ICAs from WT virgin and PP mice (*significantly different at *P* < 0.05, one way ANOVA). Bar graphs demonstrating a significant increase in diameters **(G)** and unaltered wall thickness **(H)** of arteries from CD4KO PP mice (*significantly different at *P* < 0.05, one way ANOVA).

We have previously observed that pregnancy significantly modified structural as well as functional parameters in mesenteric vessels of PP mice (Bonney et al., 2017; Gokina et al., 2021). We next turned to examine the effects of pregnancy and CD4 deficiency on the mechanical behavior of ICAs at early and late PP periods. We found that in vessels from WT mice, the 3 days and 4 weeks PP time points were associated with only a minor change in arterial distensibility at highest levels of intraluminal pressure as compared to the pre-pregnancy state (Figures 9A,B). This was also true for the distensibility of vessels from PP and virgin CD4KO mice (Figures 9C,D).

Passive lumen diameters at 80 mmHg were significantly increased in ICAs from WT 3 days and 4 weeks PP mice compared to that of ICAs from WT virgin mice (Figure 9E). Arterial wall thickness of ICAs was not modified in WT postpartum mice (Figure 9F). Although passive lumen diameters were increased in CD4KO 4 weeks PP mice, no changes were detected in vessel wall thickness at any PP period (Figures 9G,H).

## Discussion

To the best of our knowledge, this is the first study to show that pregnancy in mice can change ICAs structure and/or function PP. Although ICAs from WT mice show an outward vascular remodeling, no changes in their reactivity to PE were detected PP. In contrast, CD4 deficiency markedly enhanced ICA vasoconstrictor responses to PE at both 3 days and 4 weeks PP periods. Experimental inhibition of NO generation resulted in a significantly enhanced contractility in ICAs from WT mice. This important regulatory effect of endothelial NO in controlling ICA vasoconstriction was significantly attenuated PP in vessels from CD4KO mice.

Recent clinical studies demonstrate a significantly elevated risk for cerebral ischemia and stroke in the PP period (Kittner et al., 1996; Skidmore et al., 2001; Simon et al., 2015). Several stroke risk factors were identified, including hypertensive disorders of pregnancy, older age, infections, migraine and pre-pregnancy cardiovascular disease (Evans et al., 2011; Hovsepian et al., 2014; Leffert et al., 2015; Cunningham and LaMarca, 2018; Zambrano and Miller, 2019).

The exact mechanisms leading to PP ischemic stroke remain largely unknown. A clue to understanding this phenomenon may come from studies in rats that show a direct link between experimental stenosis of the carotid artery and reduced vasodilation of parenchymal arteries, which in turn led to impaired cognitive function (Matin et al., 2016, 2018). Additional studies in rats have revealed that significant dysfunction of small parenchymal arteries can be observed in the PP period (Cipolla et al., 2004). Further, clinical observations show that ischemic stroke can occur in patients with occlusion of ICAs (Lee J. I. et al., 2014). The carotid artery stenosis due to arterial dissections or due to spontaneous vasoconstriction in moyamoya disease is associated with an increased risk of ischemic stroke in the general population (Kittner et al., 1996; Arning et al., 1998; Lee J. I. et al., 2014; Shaik et al., 2014; Simon et al., 2015). Available clinical studies also demonstrate the link between maternal stroke and obstruction of the ICA due to arterial dissection (Kelly et al., 2014; Simon et al., 2015). *De novo* induction of moyamoya disease during early PP period was recently clinically confirmed (Miyakoshi et al., 2009; Akamatsu et al., 2014). These observations suggest that pregnancy may predispose to the occurrence of ICA stenosis followed by a significant neurological deficit and/or maternal ischemic stroke PP.

Despite the important role of ICAs in the regulation of cerebral blood flow, it remains largely unknown whether normal pregnancy can modify the function and/or structure of these vessels PP. Our current data demonstrate that ICA constrictor responses to PE were not significantly affected in WT control mice at both early (3 days) or late (4 weeks) PP periods (Figures 1, 2). On the other hand, CD4 T cell deficiency was associated with a significantly enhanced PE-induced vasoconstriction of ICAs that may result from increased sensitivity to PE or enhanced intrinsic smooth muscle contractility. To differentiate between these two mechanisms, we studied constrictor responses of ICAs to receptor-independent stimulation of vascular smooth muscle cells with high K^+^. The lack of any significant increase in K^+^-induced responses of ICAs from PP vs. virgin CD4KO (Figure 2) argues against enhanced intrinsic smooth muscle contractility as a contributor to the increased PE response observed in ICAs from CD4KO mice.

The endothelium of a mouse carotid artery can generate multiple vasodilators (NO, prostacyclin) and vasoconstrictors (endothelin) in response to chemical or mechanical stimulation (Edwards et al., 2010; Félétou et al., 2010; Thorin and Webb, 2010; Hill-Eubanks et al., 2014). In addition, endothelium-derived hyperpolarization is another mechanism contributing to ACh-induced vasodilation in carotid arteries (Grgic et al., 2009). In our study, the role of NO in the regulation of ICA function was assessed by comparing vasodilator responses to ACh and constrictor responses to PE and high K^+^ before and after inhibition of NO production by treatment with L-NNA, a global NOS inhibitor. ICAs from WT mice respond with a significant enhancement of constriction induced by receptor (PE) and non-receptor (high K^+^) stimulation after inhibition of NOS. These findings indicate that in both virgin and PP ICAs, vascular smooth muscle contraction is moderated by NO (Figures 4, 5). Although a similar augmenting effect of L-NNA was observed in arteries from CD4KO virgin mice, no changes in PE-induced constriction were detected in L-NNA-treated vessels from 3 days or 4 weeks CD4KO PP. Therefore, in CD4 deficient mice, some changes in carotid vessels induced during pregnancy and/or during the early PP period led to the reduced NO-dependent control of vasoconstriction to PE.

In resistance arteries, stimulation of alpha adrenoreceptors on vascular smooth muscle (VSM) results in generation of second messengers, e.g., inositol trisphosphate (InsP_3_) and diacylglycerol, leading to intracellular Ca^2+^ elevation and Ca^2+^ sensitization and contraction of VSMs (Brozovich et al., 2016). InsP_3_ can diffuse from the cytosol of VSM cells into ECs via myoendothelial gap junctions, stimulate localized Ca^2+^ transients and activate endothelial intermediate conductance K^+^ (IK) channels. Subsequent EC and VSM hyperpolarization moderate vasoconstriction, thus providing myoendothelial feedback regulation of the PE-evoked response (Nausch et al., 2012; Tran et al., 2012; Murphy and Sandow, 2019). In basilar arteries, the myoendothelial feedback also involves generation of NO most likely secondary to elevation of Ca^2+^ in cytosol of ECs due to Ca^2+^ diffusion beyond myo-endothelial areas and/or IK-induced hyperpolarization (Kerr et al., 2012; Murphy and Sandow, 2019). We suggest that a similar myoendothelial feedback mechanism may also operate in ICAs and restricts PE-induced sustained vasoconstriction in WT mice but may be inhibited in ICAs from PP CD4KO mice.

We found that inhibition of eNOS resulted in no change in high K^+^-induced vasoconstriction of ICAs from 4 weeks PP CD4KO mice where we detected a significant reduction in eNOS (Figures 5F, 7B). Potentiation of high K^+^-induced ICA constriction after eNOS blockade likely involves an alternative mechanism, as high K^+^ induced constriction relies on Ca^2+^ entry via voltage-gated Ca^2+^ channels with no InsP_3_ generation (Brozovich et al., 2016). This idea is supported by our finding of a much stronger enhancement of PE-induced vasoconstriction (Figure 4) compared to high K^+^-evoked responses (Figure 5) after inhibition of eNOS. Recently it has been shown that Ca^2+^ entry through voltage-gated Ca^2+^ channels can pass from VSMs to ECs and activate Ca^2+^ signaling in ECs (Garland et al., 2017). In ICAs, this endothelial Ca^2+^ rise may activate eNOS with generation of NO that moderates K^+^-induced constriction. Although somewhat controversial (Dora et al., 1997; Tran et al., 2012; Garland et al., 2017), this mechanism may provide a reasonable explanation for our findings and makes the complex area of endothelial Ca^2+^ homeostasis a potential focus for future study.

Although we observed a reduction in eNOS expression in ICAs from PP CD4KO mice, concurrent modulation of eNOS activity cannot be ruled out. It is recognized that eNOS activity in vascular ECs is regulated by multiple mechanisms (Tejero et al., 2019), including substrate and cofactor availability. In resting ECs, eNOS activity is low due to interaction with caveolin-1 (Cav-1). Ca^2+^ elevation in ECs results in the formation of Ca^2+^-calmodulin complexes that displace Cav-1, thus allowing eNOS activation and generation of NO. Additionally, eNOS activity can be stimulated by phosphorylation in response to a number of intracellular kinases, including Akt, PKA, PKC, CaMKII [reviewed in Fleming (2010) and Sutton et al. (2020)]. Other modifications, such as Glutathionylation, S-Nitrosation, and N-Acetyl glycosylation also modify enzyme activity. iNos typically is expressed in macrophages and was not expected to be differentially expressed in PP WT and CD4KO endothelium (Figures 8C,D) or in smooth muscle (Supplementary Figure 6). Studies exist suggesting that macrophages are regulated by pregnancy, with enhanced activity around the time of delivery (Norton et al., 2009; Timmons et al., 2009) and this may explain the steady decrease over PP time.

Our study demonstrates that ICAs are remarkably sensitive, regardless of CD4 deficiency, to ACh with a maximal dilator response achieved at 0.1 μM (Figure 3). NOS inhibition resulted in right-ward shift in concentration-response curves with a minimal effect on maximal vasodilation. Contribution of endothelium-derived vasodilators, other than NO, generated in response to ACh is well documented. These include prostacyclin and EDHF (Grgic et al., 2009; Edwards et al., 2010; Félétou et al., 2010). We have observed that in the vessels of 4 weeks PP WT mice, combined treatment of ICAs with L-NNA and indomethacin to block generation of endothelial NO and prostacyclin, resulted in significant preservation of vasodilation to ACh (Supplementary Figure 8). These data correlate with previously published observations in mice carotid arteries where ACh still produced near maximal vasodilation after blockade NO and prostacyclin production. In published studies, NO- and prostacyclin-independent EDHF-mediated responses were inhibited by a combined treatment with TRAM34 and UCL1684, specific inhibitors of intermediate- (IK) and small-conductance (SK) Ca^2+^-activated K^+^ channels (Feletou and Vanhoutte, 2006). We have observed a similar mechanism is responsible for EDHF-induced vasodilation in rat uterine arteries (Gokina et al., 2015).

Our data demonstrate that in both WT and CD4 deficient mice, the PP period is associated with a modest increase in ICA lumen diameters with a potential lag in the case of CD4 deficiency (Figure 8), and with minor changes in passive arterial distensibility. Published data from animal studies as well as clinical data show a significant increase in arterial distensibility in the PP mesenteric vasculature (Bonney et al., 2017; Morris et al., 2020; Gokina et al., 2021) or increased vascular compliance in PP women (Morris et al., 2015). Modest changes in carotid artery distensibility in the PP period is a novel finding demonstrating significant variations in the effect of pregnancy on the structure of resistance (mesenteric) vs. systemic (internal carotid) arteries.

In general, deficiency in T cells and B cells in mouse models is associated with differences in microvascular arteriolar baseline diameter and reactivity to vasoconstrictors and vasodilators (Leonard et al., 2011; Mukhopadhyay et al., 2020). During pregnancy this results in mid-gestational differences in heart rate and mean arteriolar pressure, thus suggesting that these cell subsets may influence systemic vascular biology in the mother both developmentally and during pregnancy (Burke et al., 2011). While evidence suggests that deficiency in a certain subset of T cells with regulatory properties (Treg) is associated with preeclampsia (Cerdeira et al., 2012; Przybyl et al., 2015) our expanding knowledge of the complexity of these T cell subsets is likely to reveal associations between this disease and several members of the CD4 family of T cells (Ahn et al., 2020; Saigusa et al., 2020; Deer et al., 2021).

CD4 T cells comprise a variety of cell types that can produce variable cytokines with both positive (e.g., angiogenesis) and negative (e.g., arteriosclerosis) effects on vascular function. CD4 T cells may influence vascular function on several levels. We hypothesized that these cells may infiltrate the vessel wall and/or perivascular tissues during pregnancy and/or PP. Indeed, we found evidence of these cells in the wall of ICAs from WT PP mice (Supplementary Figure 7), where their presence tended to be increased as compared to that observed in vessels from WT virgins. Increased blood flow/shear stress results in accumulation of CD4 T cells in the wall of mesenteric arteries (Caillon et al., 2016). Disrupted flow in WT mouse carotid arteries leads to increased expression of ICAM and VCAM (Nam et al., 2009), which could support vessel wall T cell infiltration. Carotid artery blood flow is reduced in women late pregnancy, but it is significantly increased at early postpartum (Bergersen et al., 2006; Batur Caglayan et al., 2019). Based on these observations, we speculate that increased blood flow/shear stress may act, along with systemic homeostatic expansion of CD4 T cells (Bonney et al., 2011) to promote CD4 T cell presence in the wall of ICAs PP in WT mice (Supplementary Figure 7). Other molecules, such as RANTES and CCR5 mediate T cell infiltration in animal models of hypertension (Norlander et al., 2018) and may regulate intramural CD4 T cell trafficking PP. Local production cytokines, such as IL-17 by CD4 T cells in mesenteric arteries can result in increased eNOS expression (Caillon et al., 2016). Several inflammatory cytokines (IFN-γ, TNF, IL-1β) or their combination can alter expression or activity of eNOS in HUVEC cells in a Ca^2+^-dependent manner via, for example, elevations in tetrahydrobiopterin levels (Rosenkranz-Weiss et al., 1994). Inflammatory cytokines may alter eNOS expression through activities of the transcription factor NF-κB (Lee K.-S. et al., 2014) which has both a canonical and non-canonical signaling pathway (Sun, 2011). This raises an intriguing potential link between CD4 T cells deficiency and decreased eNOS expression in 4 weeks PP ICAs (Supplementary Figure 9). Based on available data, we suggest that cytokines derived from CD4 T cells can modulate eNOS activity in ICAs by affecting endothelial Ca^2+^-signaling and/or phosphorylation state of eNOS. Inflammatory cytokines, such as TNF can also increase expression of NADPH oxidase NOX-2 in vascular cells (Bollmann et al., 2014). This could enhance vascular cell production of superoxides and in turn lead to not only decreased NO, but also decreased function of its receptor, s-guanyl cyclase (Tejero et al., 2019). Local elaboration of reactive oxygen species could also decrease function of eNOS or have other downstream effects on NO homeostasis. The dependence of T cell homeostasis on salvaged arginine also raises an intriguing possibility for competition between these and vascular cells (Wu R. et al., 2020). Exploring the impact of CD4 T cell-released molecules or other interactive effects on endothelial and VSM function in normal or disease states represents an important venue for future research.

These cells may also act systemically (both at distant sites and in the blood stream) by the production of immunomodulatory cytokines such as tumor necrosis factor and gamma interferon (Eid et al., 2009) and interleukin 10 (Kassan et al., 2011) that may influence endothelial and smooth muscle cell function, including the expression of NOS (Choi et al., 2004; Tang et al., 2005; Eid et al., 2009; Kassan et al., 2011, 2013). Systemically, CD4 T cells are also responsive to vasoactive factors, such as histamine (del Rio et al., 2012; Saligrama et al., 2014) which can in turn modify the class of CD4 response generated on exposure to antigen. It is formally possible that direct interaction between CD4 T cells and endothelial cells via molecules such as CD40 (Pluvinet et al., 2008; Hausding et al., 2013) or MHC proteins (Hermansson et al., 2010) may generate signals to both cells leading to activation or inhibition. Moreover, direct recognition by T cells of vascular smooth muscle cell antigens (Fujita et al., 2016) may also modify vascular smooth muscle cell homeostasis. How pregnancy effects these molecular interactions in the systemic arteriolar vasculature is not completely understood (Kleinschnitz et al., 2013).

CD4 T cell subsets collaborate with several cell types in the development of an immune response which may, in turn, modify vascular function (Trott et al., 2014), particularly in the PP (Bonney et al., 2017). For example, CD4 T cells help CD8 T cells in supporting the production of the CD8 T cell response to minor antigens including those expressed by the fetus and seminal fluid (Guerder and Matzinger, 1992). Cytotoxic CD8 T cells existing in the absence of CD4 T cell help or regulation by regulatory T cells, spewed on by the drivers of homeostatic expansion during pregnancy, could modulate general vascular cell function (e.g., death, apoptosis), but also affect NO homeostasis through many of the mechanisms mentioned above.

This work focused on the PP period, because it may represent a timeframe in which to learn unique features underlying the interaction between maternal vascular and immune systems. Further, understanding how this interaction signals or generates risk of subsequent cardiovascular disease, including hypertension during pregnancy is of great importance to maternal health. Some diseases of pregnancy have a unique PP phenotype both from a vascular and immune standpoint and our understanding of the PP immune system in health and in the context of specific PP disease is still evolving (Young et al., 2014; Lima et al., 2017; Aghaeepour et al., 2018; McTiernan et al., 2018; Brien et al., 2019; Coss et al., 2020; Osborne et al., 2020). The multiple layers of complexity inherent in the PP interaction between maternal immune and vascular systems is an area that is still ripe for examination in humans and animal models; the outcome of successful study will likely delineate specific mechanisms that can be utilized for diagnosis, management, and assessment of long term risk.

In conclusion, ICAs undergo PP regulation of vascular function and structure that is modified in the absence of CD4 cells and is related to basic mechanisms of vascular regulation outside of pregnancy. The examination of PP vascular function may ultimately provide both a framework and tools to enhance overall health in females.

## Data Availability Statement

The original contributions presented in the study are included in the article/Supplementary Material, further inquiries can be directed to the corresponding author.

## Ethics Statement

The animal study was reviewed and approved by the Institutional Animal Care and Use Committee of The University of Vermont (protocol X0-140).

## Author Contributions

NG and EB designed or performed all the experiments in their laboratories at The University of Vermont and wrote the manuscript. RF and ND significantlycontributed to data acquisition and analysis. KP maintained the mouse colony and contributed to data acquisition. All authors read, edited, and approved the manuscript.

## Conflict of Interest

The authors declare that the research was conducted in the absence of any commercial or financial relationships that could be construed as a potential conflict of interest.
