# Supplementary material for: Deficiency in CD4 T Cells Leads to Enhanced Postpartum Internal Carotid Artery Vasoconstriction in Mice: The Role of Nitric Oxide
Source: Front Physiol. 2021 Jun 16;12:686429. doi: 10.3389/fphys.2021.686429 (PMC8242360; doi:10.3389/fphys.2021.686429)
Supplement: Supplementary file 1 [file Data_Sheet_1.pdf]

SUPPLEMENTARY MATERIAL

DEFICIENCY in CD4 T CELLS LEADS to ENHANCED POSTPARTUM INTERNAL CAROTID ARTERY VASOCONSTRICTION in MICE: the ROLE of NITRIC OXIDE

Natalia I. Gokina[1] PhD, Rebecca I. Fairchild[1] BS, Kirtika Prakash[1]PhD, Nicole M. DeLance[2] BS, Elizabeth A. Bonney[1*], MD, MPH

[1]Department of Obstetrics, Gynecology and Reproductive Sciences, Larner College of Medicine, University of Vermont, Burlington, VT, United States

[2]Microscopy Imaging Center, Larner College of Medicine, University of Vermont, Burlington, VT, United States

***Correspondence:**

Corresponding Author

Elizabeth A. Bonney, MD, MPH

E-mail: Elizabeth.Bonney@med.uvm.edu

Short title: Postpartum carotid artery structure and function

Figure 1S

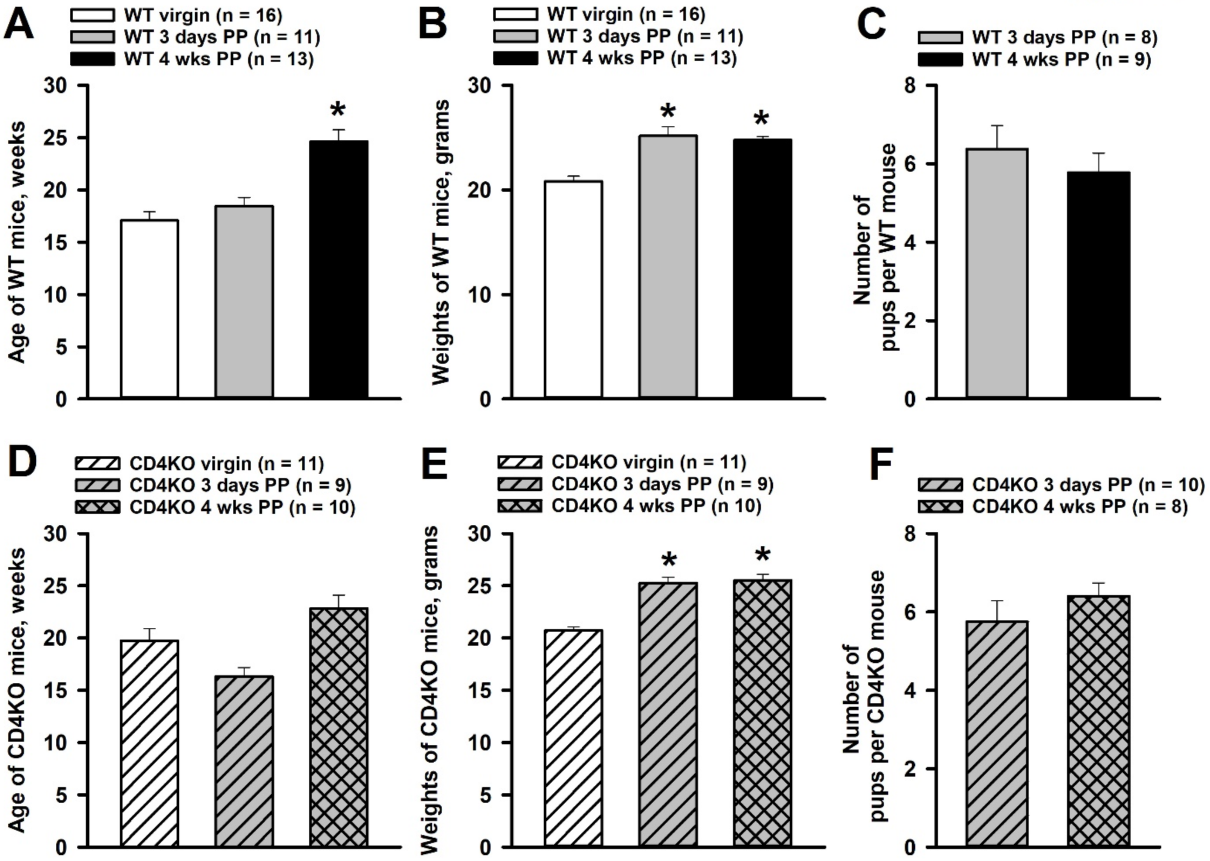

Supplemental Figure 1S. Characteristics of virgin, early (3 days) and late (4 weeks) postpartum (PP) C57BL/6 (WT) and CD4 T cell deficient (CD4KO) mice. 4 weeks PP mice were older than virgin and early PP mice **(A and D).** The weights were significantly higher in 3 days and 4 weeks PP vs. virgin mice **(B and E).** Numbers in parentheses indicate the number of mice in each studied group. **A, B, D** and **E**: one way ANOVA; **C** and **F**: unpaired t-test * Significantly different at $P<0.05$.

Figure 2S

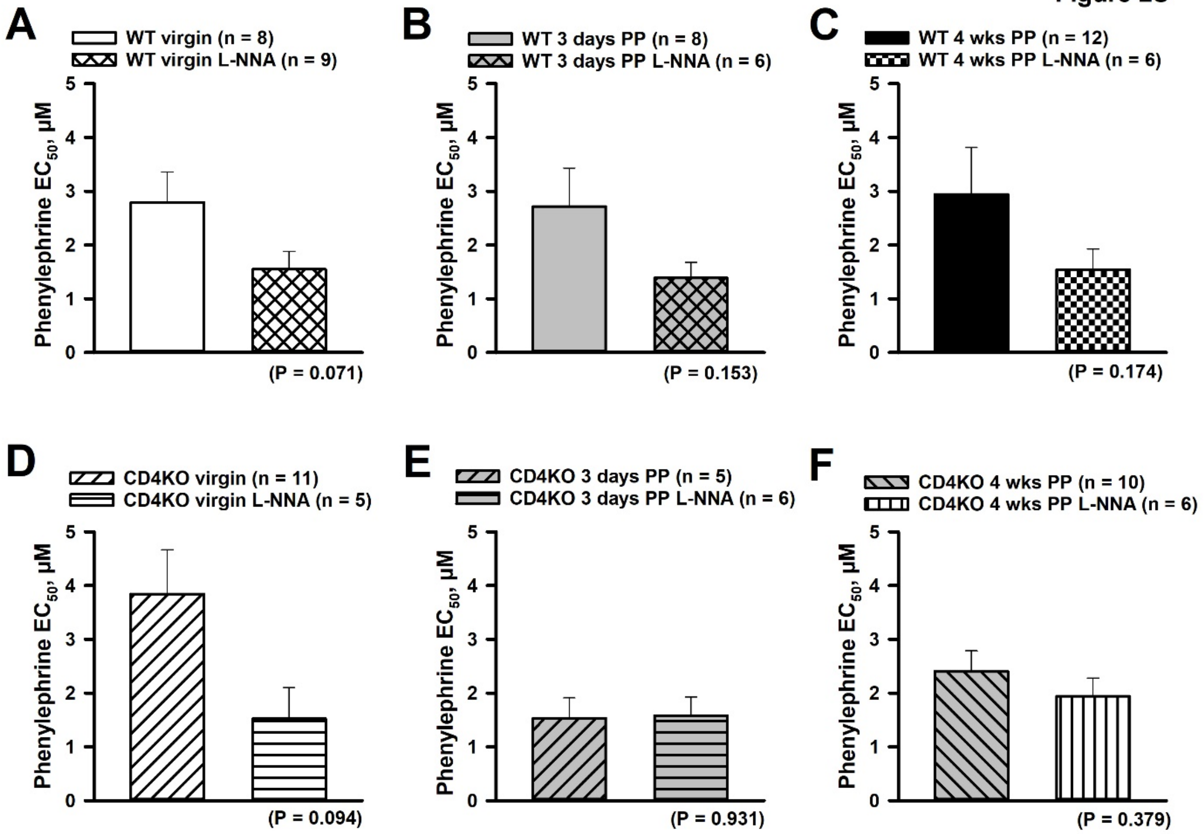

Supplemental Figure 2S. Bar graphs summarizing the effect of NOS inhibition with L-NNA on EC_{50} values for phenylephrine-induced constriction of internal carotid arteries (ICAs) from C57BL/6 (WT) and CD4 T cell deficient (CD4KO) mice. There was a trend in the reduction of EC_{50} in L-NNA treated arteries in WT virgin **(A)**, WT 3 days PP **(B),** WT 4 weeks PP **(C)** and CD4KO virgin (**D**) mice. EC_{50} were not modified in 3 days **(E)** and 4 weeks **(F)** CD4KO PP mice. (Significance of differences were defined by unpaired t-test).

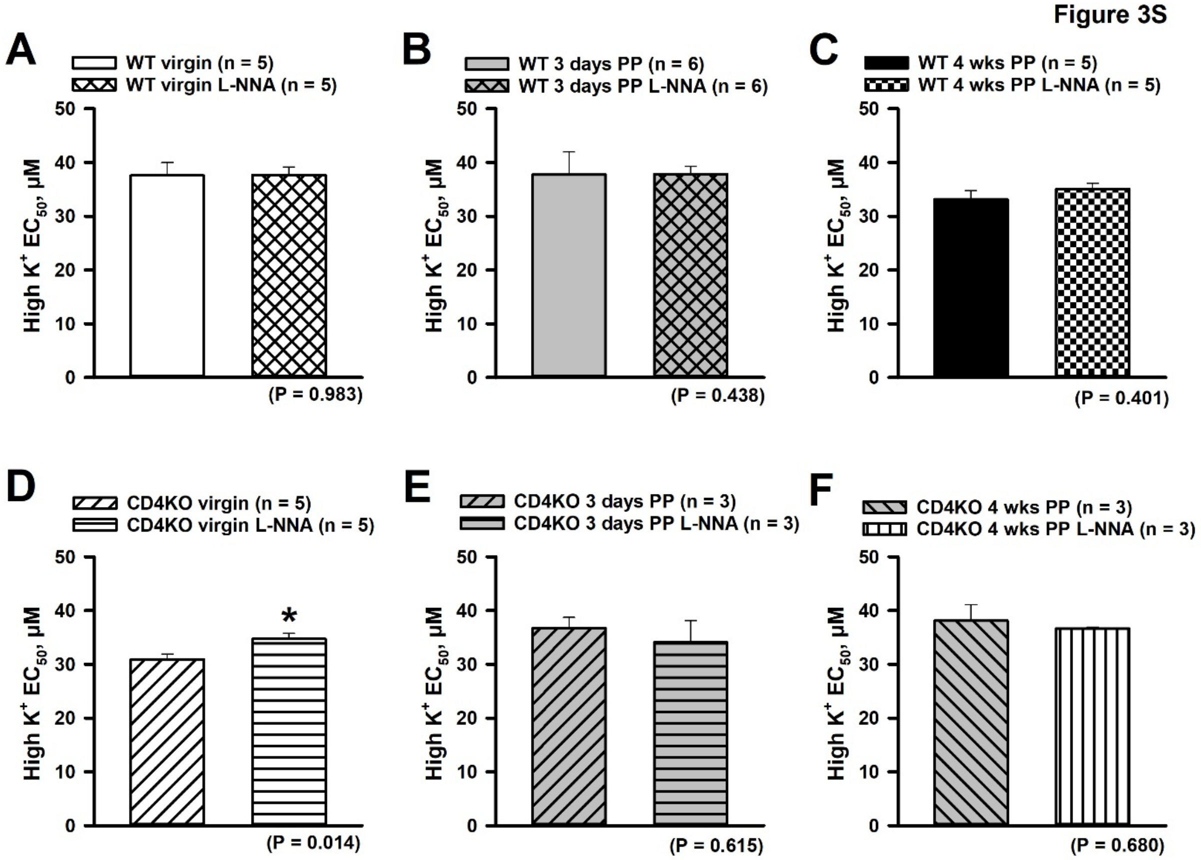

Supplemental Figure 3S. Bar graphs showing changes in the EC_{50} values for high K^+-induced constriction of internal carotid arteries from C57BL/6 (WT) and CD4 T cell deficient (CD4KO) mice before and after inhibition of NO production with L-NNA. No changes were found in EC_{50} values determined in ICAs of WT mice **(A – C).** EC_{50} calculated for ICA of CD4KO mice were increased in virgin **(D)** and were unchanged in 3 days **(E)** and 4 weeks **(F)** PP mice. P values were determined by paired t-test. Numbers in the legends for all graphs indicate the number of tested arteries. (*Significantly different at $P<0.05$, paired t-test).

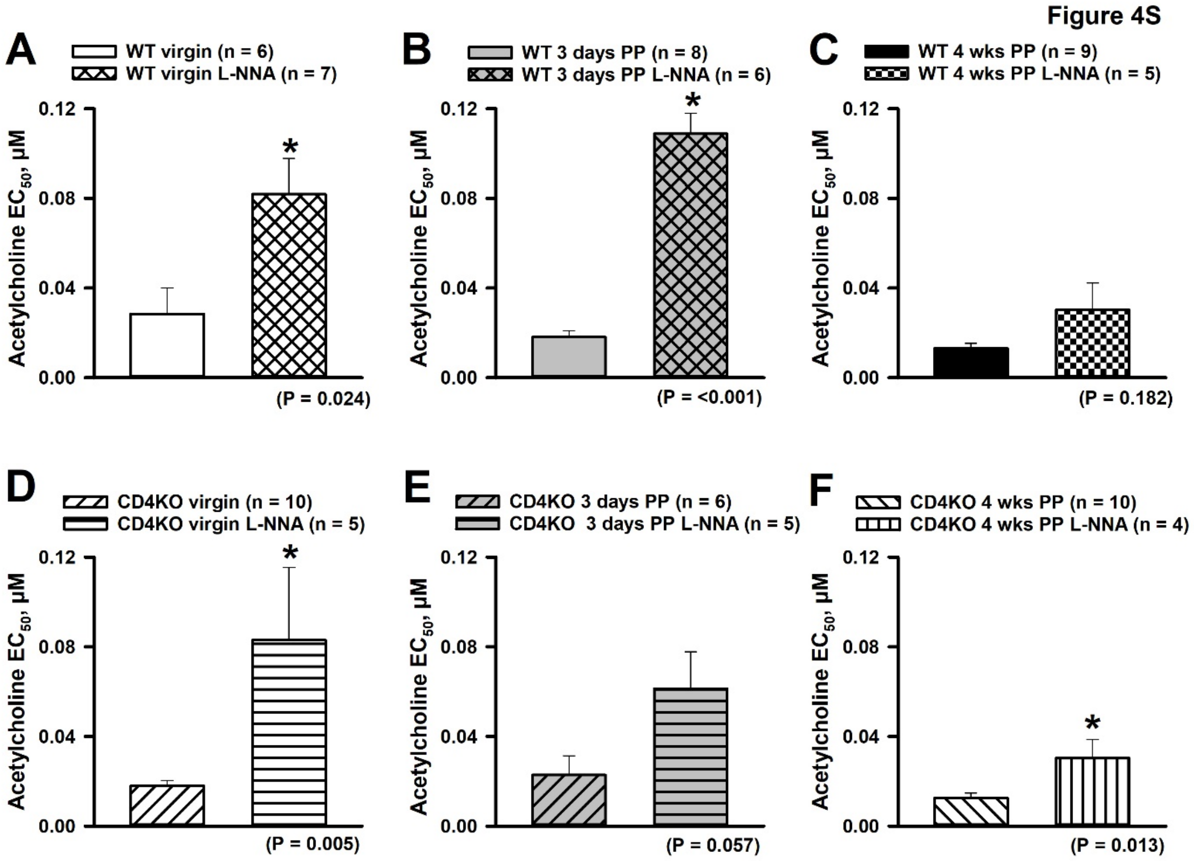

Supplemental Figure 4S. Inhibition of NO production with L-NNA resulted in increased EC_{50} values for acetylcholine-induced vasodilation of internal carotid arteries from C57BL/6 (WT) and CD4 T cell deficient (CD4KO) mice. Significant increase in ACh EC_{50} values was evident in arteries from WT virgin **(A)** and 3 days PP **(B)** as well as in CD4KO virgin **(D)** and CD4KO 4 weeks PP **(F)** mice. There was a trend in the reduction of EC_{50} in L-NNA treated arteries in WT 4 weeks PP **(C)** and CD4KO 3 days PP **(E)** mice. The numbers in graph legends indicate the number of tested arteries. (*Significantly different at $P<0.05$, unpaired t-test).

Figure 5S

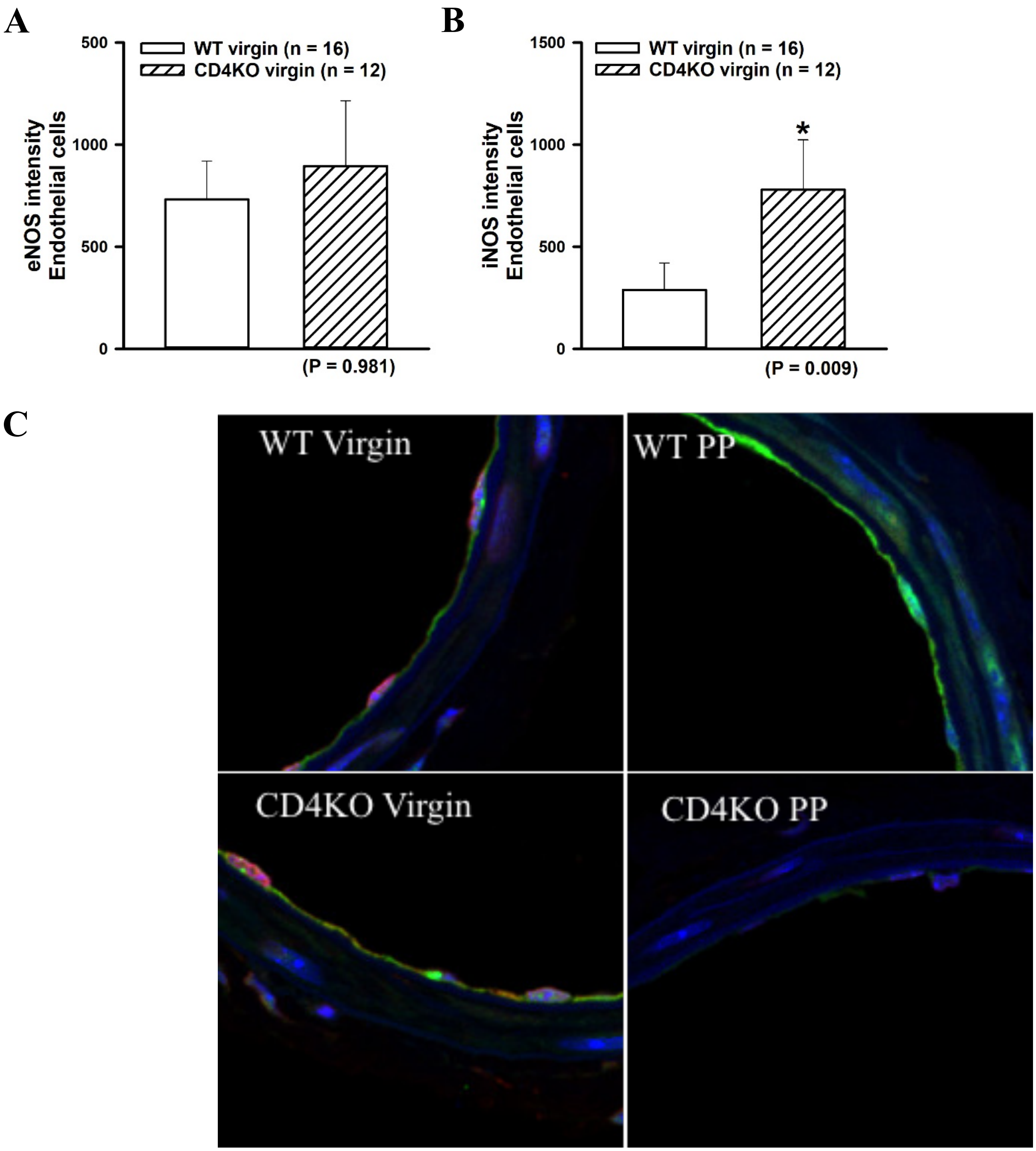

Supplemental Figure 5S. Bar graphs summarizing eNOS (**A**) and iNOS (**B**) expression in endothelial cells of internal carotid arteries from C57BL/6 wild-type (WT) and CD4 T cell deficient (CD4KO) virgin mice. iNOS expression was significantly higher in arteries from CD4KO virgin compared to WT virgin mice. The numbers in graph legends indicate the number of tested arteries. (*Significantly different at P=0.009, unpaired t-test). **(C)** Close-up view of main manuscript Figure 7 showing representative co-localization of eNOS (green) iNOS (red) and endothelial or smooth muscle cells.

Figure 6S

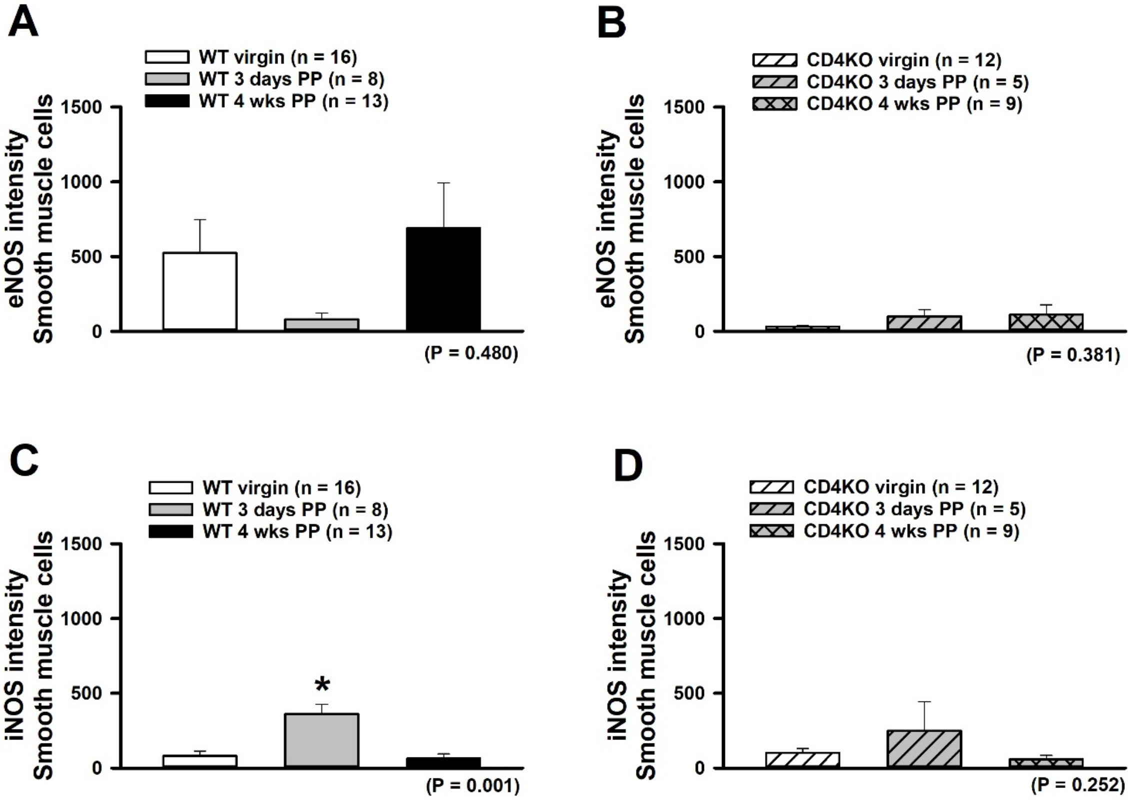

Supplemental Figure 6S. Bar graphs summarizing of eNOS (**A – B**) and iNOS (**C – D**) expression in smooth muscle cells of internal carotid arteries from C57BL/6 wild-type (WT) and CD4 T cell deficient (CD4KO) mice. iNOS expression was significantly increased in vessels from WT 3 days PP mice (**C**). The numbers in graph legends indicate the number of tested arteries. (*Significantly different at P=0.001, one way ANOVA).

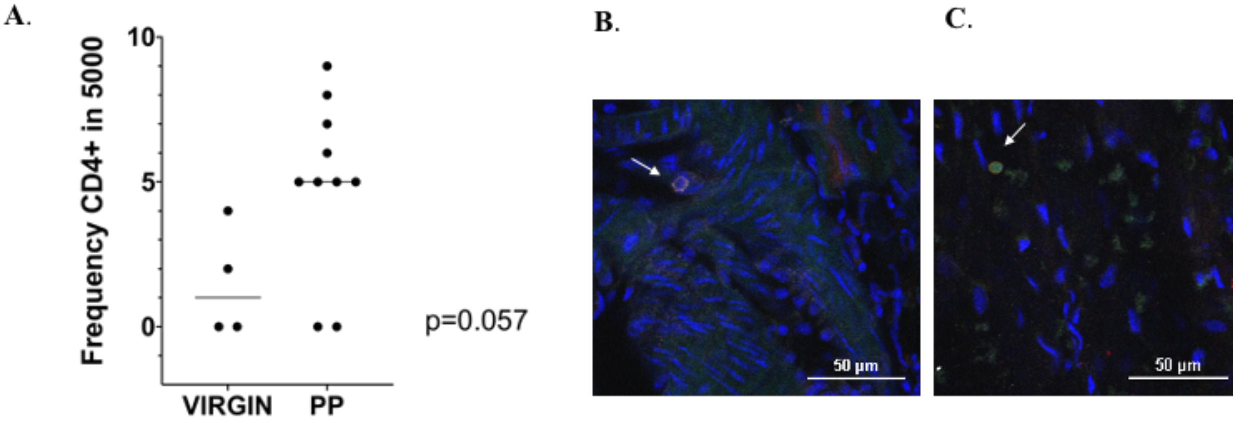

Supplemental Figure 7S. Presence of CD4 cells in internal carotid arteries from WT mice. A. Vessels from virgin or PP (3 days and 4 weeks) mice were examined by confocal microscopy of flat vessels for the presence of CD4+ cells and frequency in 5000 nuclei delineated by DAPI staining was calculated. Each symbol represents a unique section and vessel and at ~1000 cells per section were evaluated. P values were obtained using the Mann Whitney test. B. CD4 T cell in WT 3-day PP vessel wall at level of endothelial cells. C. CD8 T cells in CD4KO 3-day PP vessel.

Figure 8S

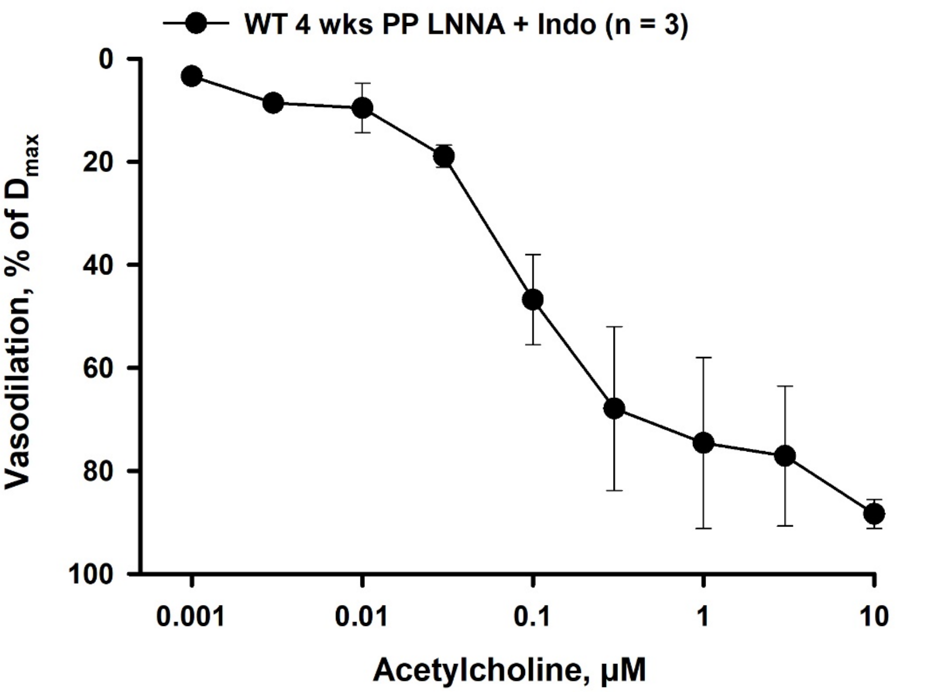

Supplemental Figure 8S. Contribution of endothelium derived hyperpolaring factor (EDHF) to acetylcholine-induced dilatation of internal carotid arteries (ICAs) from 4 weeks postpartum (PP) mice. The graph demonstrates concentration-dependent vasodilation to acetylcholine (ACh) after inhibition of nitric oxide and prostacyclin production with L-NNA (200 μM) and indomethacin (10 μM), respectively. ACh was tested in three ICAs obtained from three 4 weeks PP wild type (WT) mice.

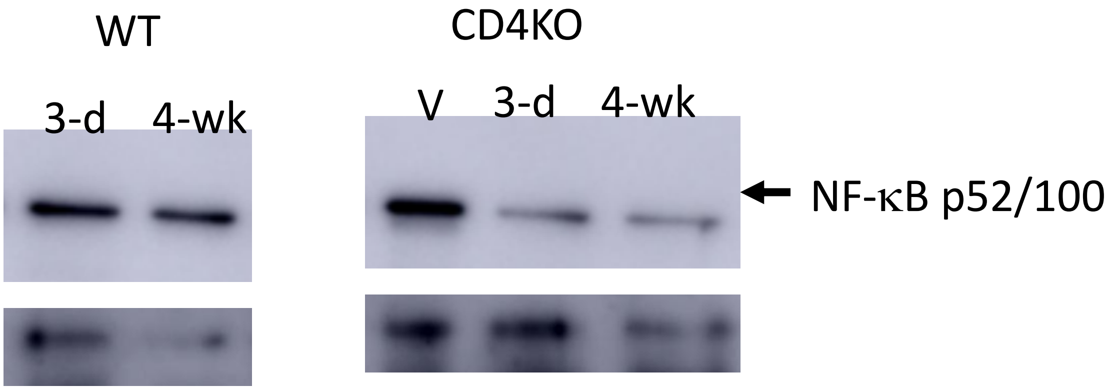

Supplemental Figure 9S. Expression of NF-κB p-100 in WT and CD4KO vessels. Internal carotid arteries from virgin (V), 3-day (3-d) and 4-week (4-wk) Postpartum mice were analyzed by Western Blot. Lower bands, Beta actin.

Supplemental table 1

Type of mice	Number of arteries	Baseline lumen diameter, µm	Type of mice	Number of arteries	Baseline lumen diameter, µm	Significance Unpaired t test
WT Virgin	11	289.6 ± 4.3	CD4KO Virgin	17	300.3 ± 3.3	P = 0.054
WT 3 days PP	14	303.4 ± 4.0	CD4KO 3 days PP	9	303.0 ± 4.8	P = 0.946
WT 4 wks PP	17	300.5 ± 3.7	CD4KO 4 wks PP	13	310.1 ± 4.1	P = 0.096

Baseline lumen diameters of internal carotid arteries from wild type (WT) and CD4 T cell deficient (CD4KO) virgin, 3 days postpartum (3 days PP), and 4 weeks postpartum (4wks PP) mice. All diameter measurements were performed at 80 mmHg in physiological salt solution (PSS) before any treatments. No significant differences in baseline lumen diameters were observed between virgin, 3 days PP and 4 wks PP WT and CD4KO mice (unpaired t-test).

Supplemental table 2

Type of mice	Number of arteries	200 µM L-NNA Baseline lumen diameter, µm	Type of mice	Number of arteries	200 µM L-NNA Baseline lumen diameter, µm	Significance Unpaired t test
WT Virgin	9	288.3 ± 5.3	CD4KO Virgin	17	289.4 ± 5.6	P = 0.899
WT 3 days PP	6	305.3 ± 7.1	CD4KO 3 days PP	9	296.8 ± 5.0	P = 0.351
WT 4 wks PP	6	312.5 ± 13.3	CD4KO 4 wks PP	13	308.5 ± 6.9	P = 0.794

Baseline lumen diameters of internal carotid arteries treated with L-NNA from wild type (WT) and CD4 T cell deficient (CD4KO) virgin, 3 days postpartum (3 days PP), and 4 weeks postpartum (4wks PP) mice. All diameter measurements were performed at 80 mmHg after 20 min of application of 200 µM L-NNA. No significant differences in baseline lumen diameters of ICAs were observed between virgin, 3 days PP and 4 wks PP WT and CD4KO mice (unpaired t-test).
